# Human and Viral microRNA Expression in Acute and Chronic HIV Infections

**DOI:** 10.3390/v16040496

**Published:** 2024-03-23

**Authors:** Elisabetta Lazzari, Gabriella Rozera, Roberta Gagliardini, Rozenn Esvan, Annalisa Mondi, Valentina Mazzotta, Marta Camici, Enrico Girardi, Andrea Antinori, Fabrizio Maggi, Isabella Abbate

**Affiliations:** 1 Laboratory of Virology, National Institute for Infectious Diseases “L. Spallanzani” IRCCS, 00149 Rome, Italy; elisabetta.lazzari@inmi.it (E.L.); fabrizio.maggi@inmi.it (F.M.); isabella.abbate@inmi.it (I.A.); 2 Clinical Department, National Institute for Infectious Diseases “L. Spallanzani” IRCCS, 00149 Rome, Italy; roberta.gagliardini@inmi.it (R.G.); annalisa.mondi@inmi.it (A.M.); valentina.mazzotta@inmi.it (V.M.); marta.camici@inmi.it (M.C.); andrea.antinori@inmi.it (A.A.); 3 AIDS Referral Center, National Institute for Infectious Diseases “L. Spallanzani” IRCCS, 00149 Rome, Italy; rozenn.esvan@inmi.it; 4 Scientific Direction, National Institute for Infectious Diseases “L. Spallanzani” IRCCS, 00149 Rome, Italy; enrico.girardi@inmi.it

**Keywords:** acute HIV infection, pathogenesis, miRNA expression, transcription

## Abstract

Human and viral microRNAs (miRNAs) are involved in the regulation of gene transcription, and the establishment of their profiles in acute (AHI) and chronic (CHI) HIV infections may shed light on the pathogenetic events related to different phases of HIV disease. Next-generation sequencing (NGS) of miRNA libraries was performed, and the reads were used to analyze miRNA differential expression in the plasma with AHI and CHI. Functional analysis was then undertaken to investigate the biological processes characterizing the two phases of HIV infection. Except for hsa-miR-122-5p, which was found in 3.39% AHI vs. 0.18% CHI, the most represented human miRNAs were similarly represented in AHI and CHI. However, when considering the overall detected miRNAs in AHI and CHI, 15 displayed differential expression (FDR *p* < 0.05). Functional analysis identified 163 target mRNAs involved in promoting angiogenesis activation in AHI versus CHI through the action of hsa-miR10b-5p, hsa-miR1290, hsa-miR1-3p, and hsa-miR296-5p. The viral miRNAs detected, all belonging to herpesviruses, accounted for only 0.014% of total reads. The present data suggest that AHI patients exhibit strong innate immune activation through the upregulation of hsa-miR-122-5p and early activation of angiogenesis. More specific investigations are needed to study the role of viral miRNAs in HIV pathogenesis.

## 1. Introduction

MiRNAs are small, non-coding, single-stranded RNAs spanning approximately 21–23 nucleotides. It is well known that miRNAs are key players in the regulation of genomic transcription associated with different human conditions, such as autoimmune, liver, and neurodegenerative diseases, as well as cancer [1]. Although miRNA-target interactions usually lead to target repression/decay, miRNAs can also stimulate the expression of target genes [2]. These small non-coding RNAs are also involved in the pathogenesis of many infectious diseases with bacterial, parasitic, and viral etiologies, like Ebola, Dengue, and Human Immunodeficiency Virus (HIV)-related diseases [3,4,5,6]. Different studies have described the possible mechanisms of action of different human miRNAs in HIV infection, which function in either promoting or suppressing viral replication. Host miRNAs may regulate HIV-1 directly, targeting either the 3′UTR of different HIV mRNAs or specific *nef* transcripts, or, more indirectly, by regulating host proteins. It has been shown that some miRNAs may prevent HIV entry, genome integration, and replication into target cells, while others facilitate viral replication or promote proviral integration and latency [1,7]. To better understand the mechanisms and pattern of miRNA expression in people living with HIV (PLWH), different populations, such as elite controllers (ECs), long-term non-progressors (LTNPs), and viremic patients have been investigated [8,9], and the response to antiretroviral treatment has also been considered [10]. These studies demonstrated that human miRNA profiles expressed in ECs and healthy donors did not significantly differ, while significant differences were found between the miRNA profiles of chronically ill patients and healthy donors. MiRNAs, especially circulating miRNAs, are used as potential biomarkers in many diseases, particularly hematological disorders, due to their high specificity and sensitivity and their easy accessibility. They are also present in many biological fluids, often displaying a sort of tissue specificity. For these reasons, their expression profiles may be used for potential diagnostic approaches [11]. In addition, antisense locked nucleic acid (LNA) inhibitors of some miRNAs are currently being studied for their efficacy in reducing HCV replication [6]. Several viruses, particularly members of the Herpesviridae family, encode their viral miRNAs [12,13,14]. Moreover, it is well known that HIV also encodes for viral miRNAs [15,16]. PLWH show a lot of viral co-infections with either viruses that establish latent infection or cause productive infection with the continuous release of viral particles in the infected host. To date, no study has compared the expression levels of human and viral miRNAs between AHI and CHI. In addition, to our knowledge no viral encoded miRNAs were described in HIV infected patients. The present study aims to establish the miRNA profiles (both human and viral) in the plasma of patients with AHI and CHI to get further insights into the pathogenetic events characterizing the different phases of HIV infection, including the possible contribution of viral co-infections.

## 2. Materials and Methods

### 2.1. Patients and Virological Characterization

Thirteen AHI subjects, from the National Institute for Infectious Diseases (INMI) observational cohort of primary infection (SIREA cohort), and five CHI subjects presenting with advanced HIV disease, all naïve to antiretroviral treatment, were evaluated at HIV serodiagnosis. Serodiagnosis of AHI patients was posed based on either the combination of an HIV Ab/Ag Combo positive and an immunoblot negative assay (Fiebig II/III stage) or an HIV Ab/Ag Combo positive and immunoblot indeterminate assay (Fiebig IV stage), according to the WHO criteria for the Western blot confirmatory assay, i.e., two *env* reactive bands. CHI patients were consecutively enrolled as they presented for clinical evaluation. Inclusion/exclusion criteria were as follows: ART-naïve HIV-infected, aged 18 years or older, newly diagnosed with HIV-1 infection within 7 days before the enrolment, presenting with confirmed AIDS-defining event, and/or CD4 cell count below 200 cells/μL. Participants with severe renal impairment (creatinine clearance < 30 mL/min), severe hepatic impairment (Child–Pugh Class C), active tuberculosis (TB), cryptococcosis, or requiring systemic cancer chemotherapy (excluding Kaposi’s sarcoma and lymphomas) were considered not eligible. Considering the AIDS-defining illnesses, three CHI subjects suffered from Pneumocystis jirovecii pneumonia (CH1, CH2, and CH4). Among these, CH1 also had progressive multifocal leukoencephalopathy, while CH4 was affected by Kaposi sarcoma and disseminated CMV disease.

HCV Ab and HBsAg/anti-HBV core antigen determinations were performed using ALINITY I HCVAb, HBsAg qualitative, and HBctot RGT, respectively (Abbott Diagnostics, Chicago, IL, USA). CMV IgG, VCA IgG, EBV IgM, EA IgG, and EBNA IgG were quantified with LIAISON^®^ kits on LIAISON^®^ XL (DiaSorin, Saluggia, Italy). HHV8 IgG were detected using an indirect immunofluorescence assay (IFA) (Euroimmun Labordiagnostika, Lubeck, Germany). Plasma HIV-1 RNA was measured by Aptima™ HIV-1 Quant Dx Assay (Hologic, Inc., San Diego, CA, USA). CMV DNA, EBV DNA, and HHV8 DNA quantification were performed on whole blood by CMV ELITE MGB, EBV ELITE MGB, and HHV8 ELITE MGB on the ELITe InGenius instrument (ELITech Group S.p.A, Turin, Italy), respectively. The SIREA study was approved by the INMI Ethical Committee on 18 February 2014. All subjects were requested to sign an informed consent form.

### 2.2. MiRNA Extraction and Reverse Transcription

Purification of cell-free total RNA, including miRNA and other small RNA, from 200 µL of plasma was performed using the miRNeasy Serum/Plasma Advanced Kit (QIAGEN, Hilden, Germany). Pre-library preparation quality control was carried out using the QIAseq miRNA Library QC PCR Panel (QIAGEN, Hilden, Germany). During the procedure, 1 µL of spike-in controls per sample were added to the lysate for monitoring the comparability and reproducibility from RNA isolation to sequencing, and 0.5 µL of UniSp6 spike-in was also added per reverse transcription reaction.

### 2.3. MiRNA Library Construction and NGS

MiRNA libraries were constructed using the QIAseq miRNA Library Kit (QIAGEN). The library preparation was performed according to the manufacturer’s protocol. Libraries quality control and quantification were carried out using the Agilent Bioanalyzer 2100 with the High Sensitivity DNA kit assay (Agilent Technologies, Santa Clara, CA, USA) and applying the size-selection mode set to tight 180 bp. When high concentrations of adapter dimers were present within the samples, library preparation was repeated by increasing the dilution of 3′ and 5′ adapters and decreasing the number of library amplification cycles. MiRNA library sequencing was performed using an Ion 540 Chip on the Ion GeneStudio S5 sequencer (ThermoFisher Scientific, Waltham, MA, USA).

### 2.4. Bioinformatics Analysis

FASTQs were obtained from the GSS5 PRIME Torrent server (ThermoFisher Scientific). CLC Genomics Workbench 23.0.5 (QIAGEN) [17] and the Biomedical Genomics Analysis plugin v. 23.1 (QIAGEN) were used to analyze the FASTQ files and to perform the statistical analysis of miRNA differential expression. Quantification analysis was carried out using the QIAseq miRNA quantification workflow (QIAGEN) with default settings. This tool allowed miRNA annotation on both human and viral reference genomes using miRBase v.22. [18]. For differential expression analysis, the QIASeq miRNA differential expression workflow (QIAGEN) was used. The workflow was set as follows: expression table: grouped on mature; test differential expression due to infection; while controlling for not set; control group: CHI. The parameters used to identify significantly differentially expressed miRNAs were a false discovery rate (FDR) with a *p*-value < 0.05 and a fold-change (FC) > 2.5.

### 2.5. MiRNA Differential Expression Analysis and Gene Ontology (GO) Enrichment Analysis

In the CLC software, the QIAseq miRNA differential expression workflow (QIAGEN) allowed for GO enrichment analysis, which can be used to investigate the biological process in which all the differentially expressed miRNAs identified were involved. The analysis was set according to the following parameters: expression table: grouped on mature; test differential expression due to infection; while controlling for not set; control group: CHI. Among the results identified as significant, we selected GO terms with a *p*-value of 0.05 and differentially expressed (DE) genes > 2. To visualize the relationships between miRNA, targets, and associated biological processes, differentially expressed miRNAs, as identified by GO Enrichment analysis, were analyzed using the Ingenuity Pathway Analysis (IPA) software, version V01-23 01 (QIAGEN) [19,20]. MiRNAs associated with each GO Term, identified in the previous analysis, have been selected. For the selected miRNAs, the ‘microRNA target filter’ tool was used to investigate the mRNA targets with the following parameter: miRNA Confidence Level: Experimentally Observed, High (Predicted). Core analysis has been performed on all the mRNA targets of the selected miRNAs. The workflow was set as follows: Core Analysis type: Expression Analysis. Since the GO enrichment analysis seemed to highlight biological processes linked mainly to angiogenesis, miRNA targets involved in this process were visually analyzed. The network was created importing the miRNAs and angiogenesis pathway into a new graph: miRNAs have been imported from the CLC dataset, while the angiogenesis process was imported from the ‘Diseases and Functions’ IPA dataset.

Overlay and Build functions have been used as follows:

- Build–Grow tool: miRNA Confidence Level: Experimentally Observed, High (Predicted); Diseases: Antimicrobial Response, Infectious Disease, Inflammatory Response.
- Overlay: analyses, datasets, and the List tool was used to import the DE analysis data onto the graph. Then, the Build–Connect tool was used to link angiogenesis to target mRNAs.

To highlight the miRNA targets involved in the angiogenesis process, all targets not linked to the investigated pathway were removed.

## 3. Results

### 3.1. Clinical and Virological Features of the Subjects

All AHI subjects were males with a median age of 46 years (IQR: 36–52), while most advanced CHI were males (four males, one female) and had a median age of 38 years (IQR: 35–47). The median (IQR) HIV-1 RNA and CD4 T cells were 6.7 (6.0–7.2) and 5.7 (5.5–6.4) log copies/mL and 425 (227–543) and 35 (25–128) cell counts/mm^3^ in AHI and CHI, respectively. All AHI and CHI subjects were anti-HCV negative and HBsAg/anti-HBV core antigen negative, with only exception of one CHI (namely, CHI 5) who was anti-HBV core antigen positive. All subjects, except for CHI 2, were anti-CMV IgG positive, with only two subjects (AHI 16 and CHI 4) having detectable CMV DNA. All subjects were anti-EBV VCA IgG, anti-EBV EBNA IgG, and anti-HSV IgG positive, with CHI 3 reacting positive for EBV DNA. Anti-HHV8 IgG was found in 3 out of 13 AHI and 4 out of 5 CHI patients; these seven subjects were also HHV8 DNA positive, thus indicating active HHV8 replication.

### 3.2. Human miRNA Analysis

The reads obtained by NGS from the plasma miRNA libraries were first aligned to the human miRNA database, providing a median number of mature miRNA sequences of 2,325,085 (IQR: 1,745,037–2,866,287) and 2,585,626 (IQR: 1,944,677–4,068,812) in AHI and CHI subjects, respectively, representing about 78% of all the obtained reads. In Table 1, the most represented miRNAs obtained in AHI and CHI, considering a cut-off of >1 of the percentage mean of the reads for each miRNA, are shown.

All of the miRNAs discussed were detected in all subjects in the two different groups. Overall, the most represented human miRNAs in both PLWH groups were similar, with only slight differences in the percentage mean reads. The only exception concerned hsa-miR-122-5p, which was found with mean detected reads of 3.39% and <1 (0.18%) in AHI and CHI subjects, respectively.

Then, the differential expression of all the human-detected miRNAs in AHI and CHI was evaluated with a statistical approach. The results of the differential expression analysis, reported in Supplemental Table S1, showed that 135 out of 2632 detected human miRNAs were statistically differentially expressed in AHI and CHI (*p* < 0.05).

**Figure 1:**
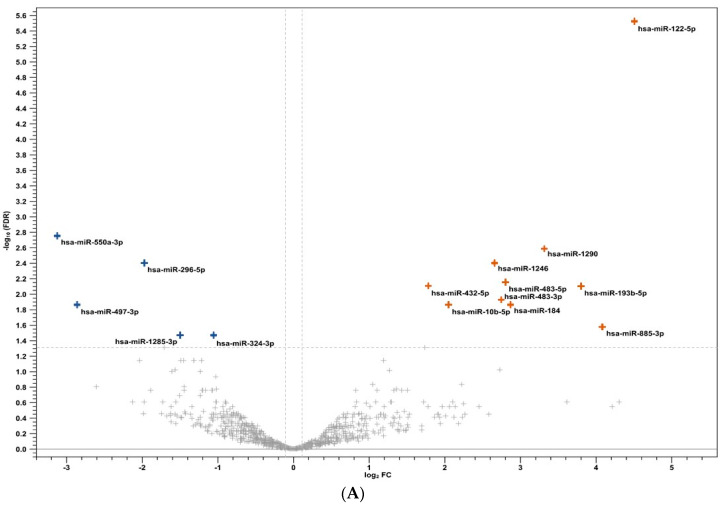

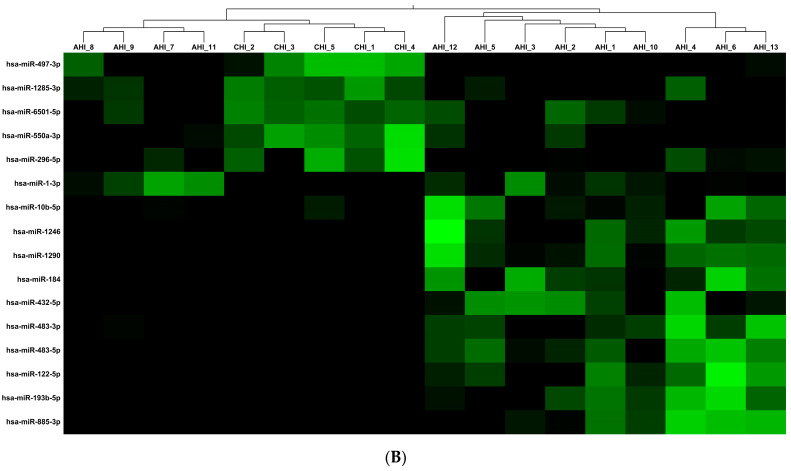
Volcano plot and heatmap of differentially expressed miRNAs in AHI versus CHI. (**A**) Volcano plot of differentially expressed miRNAs. Blue crosses indicate downregulated miRNAs in the AHI group compared to CHI, with FDR *p*-value < 0.05 and an absolute value of log FC ≥ 0.4 (equivalent to FC ≥ 2.5). Orange crosses represent miRNAs upregulated in AHI compared to CHI, with an FDR *p*-value < 0.05 and a logFC ≥ 0.4. Grey crosses represent miRNAs not statistically significant. (**B**) Heatmap of differentially expressed miRNAs with FDR *p*-value < 0.05. MiRNAs with FDR *p*-value < 0.05 and an absolute FC ≥ 2.5 are represented in rows and are listed on the left. Samples are listed above the graph and clustered with dendrograms. Different shades of green are used to represent expression levels of miRNAs. Light green represents high expression levels; black is used to represent low expression levels.

Based on these differentially expressed miRNAs, a volcano plot was constructed (Figure 1A) in which all human miRNAs upregulated and downregulated with the highest statistical difference in AHI vs. CHI (with a corrected *p*-value (FDR) < 0.05) (total of 15 miRNAs) are shown.

Additionally, in Figure 1B, a heatmap of differentially expressed human miRNAs in each AHI and CHI subject was constructed, considering only the miRNAs whose expression showed an absolute fold-change (FC) > 2.5 and a difference with a corrected *p*-value (FDR) < 0.05.

As shown in Figure 1B at the top left and bottom right of the diagram, the heatmap clustered, among AHI, into two subgroups of subjects characterized by a different framework of differently expressed human miRNAs. In particular, a larger group of 9 AHI (AHI 1, 2, 3, 4, 5, 6, 10, 12, 13) displayed a differential upregulation of 9 miRNAs (hsa-miR-10b-5, hsa-miR-1246, hsa-miR-1290, hsa-miR184, hsa-miR-432-5p, hsa-miR-483-5p, hsa-miR-483-3p, hsa-miR-122-5p, hsa-miR-885-3p) as compared with the remaining 4 AHI. On the contrary, the CHI group seemed to be more homogenous in miRNA expression, showing a coordinated upregulation of hsa-miR-497-3p, hsa-miR-1285-3p, hsa-miR-6501-5p, hsa-miR-550a-3p, and hsa-miR-296-5p in all AHI.

To get insights into the molecular and cellular mechanisms underlying these differential miRNA patterns in AHI as compared to CHI, GO Enrichment analysis (biological processes) was subsequently performed on targets of all the statistically differentially expressed miRNAs identified. In Table 2, only biological processes that included at least two differentially expressed miRNAs, with a *p* value < 0.05, are reported. (For a complete list of statistically significant GO terms associated with their associated miRNAs, see Supplemental Table S2).

**Figure 2:**
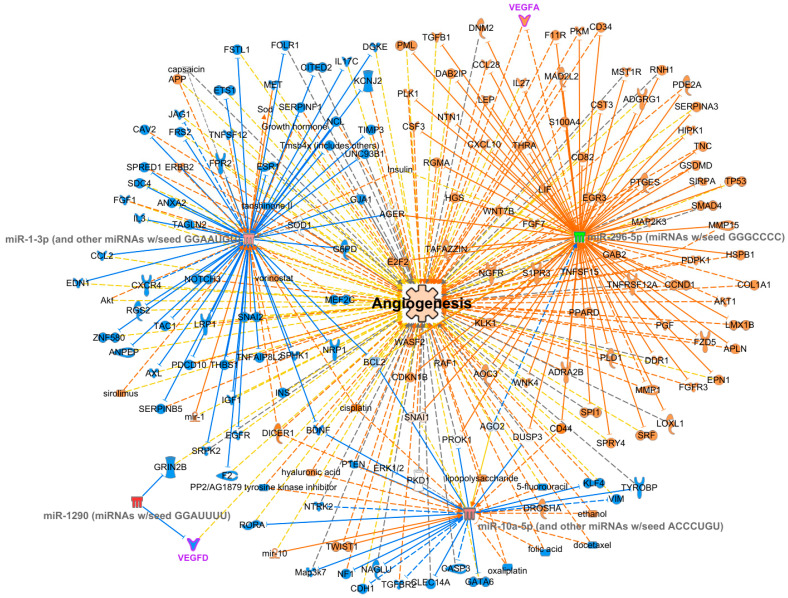
The interaction network of differentially expressed miRNAs, miRNA targets, and the biological process of angiogenesis. Representation of the interaction network of differentially expressed miRNAs, miRNA targets, and the biological process of angiogenesis, as obtained by IPA software. MiRNAs are displayed as clusters sharing the same seed sequence between round brackets. Shades of red and green are used to represent miRNA expression, according to predicted increase and decrease and expression intensity: red is for increased expression level; green is for decreased expression level. Angiogenesis biological process is displayed in the center of the network, colored in a light shade of orange since it is predicted to be activated. Relationships between miRNAs and mRNA targets and between mRNAs and angiogenesis are displayed using different types of lines: continuous lines indicate predicted direct interaction; dotted lines represent predicted indirect interaction. To show the types of relationships between miRNAs and their targets and between angiogenesis and the targets, different line colors are used: blue is for a relationship leading to inhibition; orange is for a relationship leading to activation; yellow is used to display a relationship for which findings were inconsistent with state of downstream molecules. MiRNA targets are colored using different colors linked to their predicted activity: blue is used for predicted inhibition, and orange is used for predicted activation. MiRNAs and mRNA targets are displayed with different shapes according to their biological nature (i.e., miRNAs, receptors, cytokines, enzymes, etc.). VEGFD and VEGFA are highlighted in pink as they are the main players of angiogenesis.

As shown in Table 2, 4 out of 5 GO terms are involved in vascular endothelial growth factor (VEGF) signaling and, hypothetically, the angiogenesis phenomenon. The GO Term 0051272 corresponded to a term defined as obsolete (not a useful grouping class since it mixed both cell and cellular component terms). For this reason, this GO term was not considered for subsequent analysis, including the visualization of the interactions using the IPA software (core analysis). For this analysis, the four DE miRNAs identified in the GO Enrichment analysis were selected, together with all their target mRNAs (1437). The only filter applied in the software computation concerned the miRNA confidence level selected (experimentally observed and highly predicted). In the first analysis, without search restrictions, we found that the four miRNAs were mainly involved in the cardiac hypertrophy pathway and several tumors. As a matter of fact, of the 1437 mRNA targets, 163 were involved in the angiogenesis phenomenon, with a *p*-value of 6.67 × 10^−9^. In the second analysis, we imposed “Antimicrobial Response”, “Infectious Disease”, and “Inflammatory response” filters in the ‘Disease and Functions’ section. In Figure 2, an additional representation on how the previously identified miRNAs and their predicted targets in angiogenesis is provided. In more detail, the downregulation of miR-296-5p caused a decrease in the inhibition of target mRNAs, thus promoting activation of angiogenesis, whereas the upregulation of the other miRNAs, i.e., miR-10b-5p (belonging to the miRNA10a-5p cluster), miR-1290, and miR-1-3p caused an inhibition of the targets, favoring the activation of angiogenesis.

### 3.3. Viral miRNA Analysis in Plasma from AHI and CHI Subjects

Among the non-aligned reads to the human miRNA database, 0.014% aligned to the viral miRNA database. Considering also in this case, only mature human miRNAs were detected. Table 3 shows the complete list of all the mature viral miRNAs found by NGS in the plasma samples from AHI and CHI subjects.

Only miRNAs of the Herpesvirus family were detected based on the alignment to the selected miRNA database. EBV miRNAs are more represented in CHI as compared to AHI, with ebv-miR-BART7-3p detected in 3/5 CHI compared to 1/13 AHI plasma samples, which was also reaching statistical significance for the difference (*p* = 0.044 in Fisher exact test). On the contrary, the different expression of ebv-miR-BART2-5p, ebv-miR-BART-8-5p, and ebv-miR-BART-19-3p detected in two CHI and in none of the AHI subjects did not reach statistical significance (*p* = 0.065 in Fisher exact test). Seven different kshv-miRNAs were detected in three AHI and one CHI samples. The CHI patient expressing kshv-miR-K12-6-3p, kshv-miR-K12-8-3p, and kshv-miR-K12-10a-3p was affected by Kaposi sarcoma. In addition, 99.78% of CMV miRNA reads were found in two AHI, particularly in one subject who suffered from a concomitant primary CMV and HIV infection, accounting for 447 out of 448 total AHI reads. The expression of hcmv-miR-US25-2-5p was detected in one CHI sample obtained from a patient with CMV disease. No mature HIV-1 miRNAs in either AHI or CHI were detected by this experimental approach.

## 4. Discussion

In the present study, an unbiased characterization of human and viral miRNAs was performed using the NGS of miRNA libraries obtained from plasma samples of acute and advanced chronic HIV subjects naïve to antiviral treatment. The NGS approach allowed us to get insights into the miRNAs present in the plasma of PLWH samples without a pre-selection of some specific miRNAs. Moreover, the quantitative determination of each detected miRNA enabled us to perform a statistical differential analysis of the expression of the different miRNAs detected in acute and chronic phases of the HIV infection. Starting from these data, a functional analysis of the possible involved biological process implied by the expression of these miRNAs was undertaken. Most previous studies focused on PWLH considered only pre-determined sets of miRNAs: for example, Cuesta-Sancho et al. studied a few human miRNAs chosen from the literature, and Biswas et al. used a commercially pre-formed array of 372 human miRNAs [21,22]. In our study, the NGS approach, which is able to detect any possible miRNA recognizable by an updated database with a very high sensitivity, highlighted that the six most represented miRNAs, representing 31.2% and 33.1% of the total miRNA reads in the plasma samples of AHI and CHI, respectively, were the same among the two groups. Among these miRNAs, hsa-miR-16-5p and hsa-miR-223-3p were already mentioned by Biswas et al. as miRNAs with different expression between acute HIV subjects and healthy donors [23]. Hsa-miR-223 was found to be downregulated in HIV subjects naïve to treatment concerning healthy donors [10]. In addition, hsa-miR-16-5p and hsa-miR-126-3p were shown to be associated with viral replication and ART failure [24]. Hsa-let-7i-5p was found over-expressed in chronically infected subjects compared to ECs and HIV-negative individuals [9]. In general, the hsa-let-7 family is involved with host immunity, specifically with toll-like receptors that mediate cytokine expression during infections [25].

In our study, hsa-miR-122-5p was quantitatively the most differentially expressed miRNA in AHI versus CHI (4.5 log fold change higher in AHI vs. CHI). The hsa-miR-122 is the most common miRNA found to date and interacts with several different sequences that participate in a wide variety of biological functions and play a critical role in various infectious diseases, including viral, bacterial, and parasitic infections. It has been shown that hsa-miR-122 may control apoptosis, antioxidant defence, and the immunological response, influencing the innate immune response to viral infections. For example, hsa-miR-122 amplifies the IFN-mediated innate immune response by regulating genes that impact the phosphorylation levels of STAT3. On the other hand, hsa-miR-122 has been shown to increase the replication of some RNA viruses, such as Flavivirus, Coronavirus, and Picornavirus, by binding to the 5′UTR of viral RNA and increasing the translation of viral RNA [26,27] The deregulation of hsa-miR-122, which takes place during viral infection, may have significant implications for both disease progression and treatment. Two potent antisense-locked nucleic acid (LNA) inhibitors of hsa-miR-122 (Miravirsen and RG-10) demonstrated efficacy in reducing HCV replication. [6]. Higher levels of circulating hsa-miR-122 have been detected in HIV/HCV co-infected patients than in HIV mono-infected individuals [28]. In the HIV/HCV coinfections, the *vpr*-encoded protein of HIV upregulates the expression of hsa-miR-122 and stimulates HCV 5′ UTR activity, providing new evidence for how HIV interacts with HCV during HIV/HCV co-infections [29]. In addition, a direct effect of HIV on the liver, probably mediated by an up-regulation of hsa-miR-122-5p and hsa-miR-193b-5p, has been shown by Franco et al. [30]. In our study population, no subject was anti-HCV positive, thus the differential expression of hsa-miR-122 may not be attributed to a different percentage of HCV-positive patients in the two PLWH groups. Hsa-miR-122-5p and hsa-miR-1290, the latter known to participate in HIV latency [31], were upregulated in AHI versus CHI, and they were present in the major subgroup of AHI patients highlighted by the heatmap analysis. To note, no viro-immunological characteristic (HIV-1 RNA load, CD4 T cell counts, CD4/CD8 ratio, and viral co-infections) allows to differentiate the two subgroups of AHI patients.

Other miRNAs were differentially expressed in AHI versus CHI. The first was hsa-miR-550a-3p, which inhibits the growth of vascular smooth muscle cells [32], and the second was hsa-miR-296-5p, known to be a tumor suppressor and involved in cell growth, neuronal differentiation, and survival [33]. This latter miRNA was downregulated in HIV patients [34,35]. This is in accordance with our data, which report that hsa-miR-296-5p is downregulated in AHI versus CHI samples. The last miRNA downregulated in AHI patients was hsa-miR-324, previously reported to be involved in the etiopathogenesis of cancer disorders, cardiac diseases, Parkinson’s diseases, infectious diseases, and, particularly, in HIV lipodystrophy [36]. Hsa-miR-324 was also found to be inversely correlated with the CD4/CD8 ratio and CD4 T cell counts [37].

The functional analysis, conducted considering the overall differential miRNA expression, revealed the differential regulation of VEGF and the related phenomenon of angiogenesis as the main cellular process differently activated between AHI and CHI patients. According to this evidence, VEGF-A concentrations were found higher in the sera of HIV-1-infected patients than in those of uninfected individuals [38]. VEGF is known to be responsible for vascular leakage and proliferation of vascular endothelial cells, which are both hallmarks of AIDS-associated vasculopathy [38]. The different miRNA expression profiles of AHI versus CHI, highlighted in the present article, suggest that AIDS-associated vasculopathy may begin early during HIV infection. The functional analysis conducted on our data foresees the upregulation of some pro-angiogenic factors (e.g., VEGF) from the early stages of the natural history of HIV infection.

However, using in vitro or animal models would probably clarify specific single aspects dictated by the involvement of the different miRNAs in some cellular functions, such as angiogenesis.

Also, some viral miRNAs are known to influence the homeostasis of endothelial cells, such as some HHV-8 (kshv)-encoded miRNAs. In our study population, among the seven kshv-miRNAs detected, we found kshv-miR-K12-11 and kshv-miR-K12-6. Both of them have been previously reported to induce endothelial cell reprogramming, acting in concert with a lymphatic endothelial cell-specific transcription factor, the musculoaponeurotic fibrosarcoma oncogene homolog [39]. In the plasma of the CHI subject with Kaposi sarcoma, we found kshv-miR-K12-6-3-p, kshv-miR-K12-8-3p, and kshv-miR-K12-10a-3p. Other viral miRNAs, detected in our HIV subjects, seemed to be associated with the evasion from the immune response, i.e., ebv-miR-BART7-3p, which is known for its anti-viral interferon lambda (IFNL) 3 action [40]. Regarding hcmv-miRNAs, many reads were found in two HIV AHI samples and in one CHI sample: in particular, one out of the two AHI subjects suffered from a concomitant CMV primary infection, while the CHI subject showed a disseminated CMV clinical disease. In the plasma of this CHI, we found hcmv-miR-US25-2-5p, which is the most abundantly expressed hcmv-miRNAs in our PLWH. This miRNA is known to inhibit HCMV replication [41] and to favor latency. Importantly, no HIV-encoded miRNAs were revealed by the NGS approach used in our study. This could be due to the fact that viral miRNAs are more recently discovered and the current databases for their recognition by NGS data have difficulty being updated promptly. For example, the database used in the present study could not detect some recently discovered HIV-encoded miRNAs described in [42].

## 5. Conclusions

In conclusion, the miRNA expression in the plasma of AHI and CHI subjects was investigated by using the unbiased NGS approach, revealing a massive activation of molecules related to innate immune response and, more importantly, the early activation of some pathogenetic phenomena, previously known to be associated only with late stages of HIV infection, such as angiogenesis. The differential pattern of miRNA expression may impact disease staging diagnostics but, more effectively, the awareness of these early events in HIV disease could focus on possible timely intervention to try to slow down the progression of specific pathogenetic aspects. This study, although it has the limitation of having been carried out on a few samples of two groups composed of a different number of subjects, provided an overview of miRNA expression during HIV infection, showing, as expected, an overexpression of human over viral miRNAs. Among viral miRNAs, those encoded by Herpesviruses were the only ones detected, probably for their ubiquity, diffusion, and their capacity to establish long-term infection in humans, alternating latency with productive viral replication phases. Human herpesviruses display a lot of encoded miRNAs that regulate the expression of their genes, host genes, or both. Currently, in miRNA databases, more than 140 miRNAs out of the about 200 total viral miRNAs identified encoded by alpha, beta, and gamma herpesviruses are deposited [14,43]. Especially for viral miRNAs encoded by RNA viruses, which are more recently discovered than those encoded by DNA viruses, it is possible that the databases used for viral miRNAs detection are not extremely up to date. For both these reasons, to study in even more detail the contribution of viral miRNAs in the pathogenesis of HIV infection, including the impact of co-infections, both selective experimental designs, such as specific RT-PCR and the identification of still unknown viral miRNAs based on the alignment of the reads obtained by NGS with the reference viral genomes will be necessary.

## Figures and Tables

**Table 1:** Prevalence of the most abundant human miRNAs in plasma samples from AHI and CHI.

Human Mature miRNA	AHI Mean %	CHI Mean %
hsa-miR-16-5p	8.82	10.99
hsa-let-7f-5p	5.57	4.42
hsa-miR-126-3p	5.41	5.19
hsa-let-7i-5p	4.28	4.69
hsa-let-7a-5p	3.70	3.51
hsa-miR-223-3p	3.44	4.33
hsa-miR-122-5p	3.39	<1 (0.18)
hsa-let-7b-5p	2.55	2.95
hsa-miR-142-3p	2.30	3.09
hsa-miR-21-5p	2.07	2.47
hsa-miR-103a-3p	2.02	1.61
hsa-miR-199a-3p	2.02	1.87
hsa-miR-146a-5p	2.00	2.00
hsa-miR-26a-5p	1.81	2.34
hsa-miR-26b-5p	1.73	1.73
hsa-miR-191-5p	1.64	1.06
hsa-miR-221-3p	1.02	1.37
hsa-miR-92a-3p	1.5	1.04

**Table 2:** GO enrichment analysis (biological processes) of all differentially expressed miRNAs in AHI versus CHI. Biological processes with a significant *p*-value < 0.05 and differentially expressed (DE) genes > 2 are included. Detected Genes column represents the number of detected genes in the analysis. DE Genes column represents the number of differentially expressed detected genes during the analysis. DE Genes (Names) column represents the RNACentral database identifier of the differentially expressed genes in the analysis. MiRNAs column represents the miRNAs found using RNA Central database identifier.

GO Term	Description	Detected Genes	DE Genes	DE Gene (Names)	MiRNAs (Names)	*p*-Values
0030947	regulation of the VEGF receptor signaling pathway	4	2	URS00001C3AC1_9606, URS000058760A_9606	hsa-miR-296-5p, hsa-miR-10b-5p	0.0045
0030949	positive regulation of the VEGF receptor signaling pathway	4	2	URS00001C3AC1_9606, URS000058760A_9606	hsa-miR-296-5p, hsa-miR-10b-5p	0.0046
0090287	regulation of cellular response to growth factor stimulus	20	3	URS00001C3AC1_9606, URS00001DC04F_9606, URS000058760A_9606	hsa-miR-296-5p, hsa-miR-1-3p, hsa-miR-10b-5p	0.0150
0051272	positive regulation of cellular component movement	40	4	URS00001C3AC1_9606, URS00001DC04F_9606, URS000043F369_9606, URS000058760A_9606	hsa-miR-296-5p, hsa-miR-1-3p, hsa-miR-1290, hsa-miR-10b-5p	0.0166
1903672	positive regulation of sprouting angiogenesis	22	3	URS00001C3AC1_9606, URS00001DC04F_9606, URS000058760A_9606	hsa-miR-296-5p, hsa-miR-1-3p, hsa-miR-10b-5p	0.0198

**Table 3:** Detection of viral miRNAs in AHI and CHI.

Viral miRNA	Virus	AHI Positive/Total Subjects	CHI Positive/Total Subjects	Total Counts
ebv-miR-BART11-3p	EBV	0/13	1/5	1
ebv-miR-BART7-3p		1/13	3/5	11
ebv-miR-BART9-3p		0/13	1/5	1
ebv-miR-BART2-5p		0/13	2/5	2
ebv-miR-BART6-3p		0/13	1/5	1
ebv-miR-BART15		0/13	1/5	2
ebv-miR-BART8-5p		0/13	2/5	3
ebv-miR-BART12		0/13	1/5	1
ebv-miR-BART19-3p		0/13	2/5	2
ebv-miR-BART3-5p		0/13	1/5	2
ebv-miR-BART17-5p		0/13	1/5	1
ebv-miR-BART10-3p		1/13	0/5	1
kshv-miR-K12-6-3p	Kaposi’s Sarcoma-associated Herpesvirus (HHV8)	3/13	1/5	7
kshv-miR-K12-8-3p		1/13	1/5	2
kshv-miR-K12-10a-3p		2/13	1/5	6
kshv-miR-K12-4-3p		2/13	0/5	1
kshv-miR-K12-5-3p		1/13	0/5	1
kshv-miR-K12-12-3p		1/13	0/5	1
kshv-miR-K12-11-3p		1/13	0/5	1
hcmv-miR-US25-2-5p	CMV	2/13	1/5	80
hcmv-miR-US25-2-3p		2/13	0/5	63
hcmv-miR-US25-1-5p		1/13	0/5	115
hcmv-miR-UL112-3p		1/13	0/5	3
hcmv-miR-US29-3p		1/13	0/5	1
hcmv-miR-US33-5p		1/13	0/5	13
hcmv-miR-UL59		1/13	0/5	2
hcmv-miR-UL22A-5p		1/13	0/5	53
hcmv-miR-UL22A-3p		1/13	0/5	8
hcmv-miR-US33-3p		1/13	0/5	2
hcmv-miR-US25-1-3p		1/13	0/5	13
hcmv-miR-UL36-5p		1/13	0/5	50
hcmv-miR-US5-1		1/13	0/5	9
hcmv-miR-US4-5p		1/13	0/5	12
hcmv-miR-US29-5p		1/13	0/5	9
hcmv-miR-US22-5p		1/13	0/5	7
hcmv-miR-UL36-3p		1/13	0/5	9

Total counts are the number of reads per mature miRNA identified.

## Data Availability

Data are contained within the article and Supplementary Materials.

## Supplementary Materials

The following supporting information can be downloaded at: https://www.mdpi.com/article/10.3390/v16040496/s1, Table S1: Differentially expressed miRNAs in AHI versus CHI (*p*-value < 0.05). Table S2: GO Enrichment Analysis (*p*-value < 0.05).
