# Supplementary material for: Human and Viral microRNA Expression in Acute and Chronic HIV Infections
Source: Viruses. 2024 Mar 23;16(4):496. doi: 10.3390/v16040496 (PMC11054094; doi:10.3390/v16040496)
Supplement: Supplementary file 1 [file viruses-16-00496-s001.zip › viruses-2916452-supplementary.pdf]

Supplementary Table S1. Differentially expressed miRNAs in AHI versus CHI (p-value< 0.05).

Name	Identifier	Max group mean	Log_2 fold change	Fold change	p-value	FDR p-value	Bonferroni
hsa-miR-122-5p	URS00003380CC_9606	42069.692	4.512	22.824	3E-09	3E-06	5E-06
hsa-miR-550a-3p	URS00001F14FE_9606	9.200	-3.120	-8.696	4E-06	0.002	0.006
hsa-miR-1290	URS000043F369_9606	50.462	3.315	9.950	8E-06	0.003	0.012
hsa-miR-1246	URS000028C188_9606	169.385	2.656	6.304	2E-05	0.004	0.027
hsa-miR-296-5p	URS00001C3AC1_9606	48.600	-1.973	-3.925	2E-05	0.004	0.032
hsa-miR-483-5p	URS000003575B_9606	107.846	2.805	6.990	4E-05	0.007	0.067
hsa-miR-432-5p	URS00001C406A_9606	3887.077	1.783	3.440	6E-05	0.008	0.095
hsa-miR-193b-5p	URS00000E1DC5_9606	10.615	3.801	13.935	6E-05	0.008	0.101
hsa-miR-483-3p	URS00000EA063_9606	16.692	2.747	6.712	1E-04	0.012	0.170
hsa-miR-497-3p	URS000007D64A_9606	5.800	-2.854	-7.230	2E-04	0.014	0.237
hsa-miR-10b-5p	URS000058760A_9606	153.846	2.053	4.150	2E-04	0.014	0.245
hsa-miR-184	URS0000543D82_9606	14.692	2.873	7.326	2E-04	0.014	0.262
hsa-miR-885-3p	URS00003F4546_9606	5.923	4.087	16.997	3E-04	0.026	0.546
hsa-miR-1285-3p	URS0000399545_9606	21.200	-1.490	-2.809	5E-04	0.033	0.746
hsa-miR-324-3p	URS0000D50706_9606	190.400	-1.058	-2.083	0.001	0.035	0.840
hsa-miR-6501-5p	URS000075C286_9606	13.800	-1.709	-3.269	0.001	0.049	1
hsa-miR-1-3p	URS00001DC04F_9606	1301.077	1.741	3.342	0.001	0.049	1
hsa-miR-3605-3p	URS00000A9FB9_9606	27.538	1.197	2.293	0.001	0.072	1
hsa-miR-542-5p	URS000050C722_9606	42.600	-1.316	-2.489	0.002	0.072	1
hsa-miR-500a-5p	URS000039A052_9606	19.000	-1.455	-2.741	0.002	0.072	1
hsa-miR-326	URS00000A939F_9606	1319.400	-1.215	-2.322	0.002	0.072	1
hsa-miR-9983-3p	URS0000D4FF80_9606	7.200	-2.029	-4.082	0.002	0.072	1
hsa-miR-29a-5p	URS0000076995_9606	28.000	-1.485	-2.798	0.002	0.072	1
hsa-miR-1307-5p	URS00000EEF5F_9606	892.600	-1.558	-2.945	0.002	0.095	1
hsa-miR-885-5p	URS0000246356_9606	7.000	2.733	6.647	0.002	0.095	1
hsa-miR-125b-5p	URS0000209905_9606	726.615	1.274	2.418	0.003	0.096	1
hsa-miR-641	URS000039D790_9606	58.800	-1.226	-2.339	0.003	0.100	1
hsa-miR-3127-3p	URS0000292363_9606	7.200	-1.605	-3.042	0.003	0.100	1
hsa-miR-106a-5p	URS00003FE4D4_9606	478.000	-1.021	-2.029	0.003	0.117	1
hsa-miR-28-3p	URS00001799A3_9606	3947.400	1.048	2.068	0.005	0.148	1
hsa-miR-4516	URS00000BF7F9_9606	6.615	2.226	4.678	0.005	0.148	1
hsa-miR-18b-5p	URS00004565E5_9606	13.800	-1.445	-2.722	0.005	0.159	1
hsa-miR-4697-3p	URS00003685CF_9606	12.800	-2.604	-6.081	0.005	0.159	1
hsa-miR-6764-5p	URS000075AAD9_9606	13.385	1.367	2.579	0.006	0.169	1
hsa-miR-4286	URS0000353492_9606	165.000	-1.020	-2.027	0.006	0.169	1
hsa-miR-766-3p	URS00001012BC_9606	427.800	-1.158	-2.232	0.006	0.175	1
hsa-miR-193a-5p	URS0000367985_9606	84.077	1.432	2.697	0.007	0.176	1
hsa-miR-377-3p	URS000007F792_9606	210.600	-1.199	-2.296	0.007	0.177	1
hsa-miR-940	URS000041FA2C_9606	18.600	-1.444	-2.720	0.007	0.177	1
hsa-miR-6515-3p	URS000075BDA4_9606	9.600	-1.197	-2.293	0.007	0.177	1

hsa-miR-100-5p	URS000040D674_9606	22.692	1.507	2.842	0.007	0.177	1
hsa-miR-409-3p	URS00002915C8_9606	4477.000	0.823	1.769	0.008	0.177	1
hsa-miR-22-3p	URS0000096022_9606	7567.000	-1.066	-2.093	0.008	0.177	1
hsa-miR-3176	URS00004AAAFF_9606	5.000	-1.889	-3.704	0.008	0.177	1
hsa-miR-2355-3p	URS00003804D8_9606	344.800	-1.076	-2.108	0.008	0.177	1
hsa-miR-191-5p	URS00005C2E31_9606	56250.200	1.107	2.154	0.008	0.177	1
hsa-miR-5187-5p	URS000036B845_9606	105.077	1.328	2.511	0.008	0.177	1
hsa-miR-1537-3p	URS000010F787_9606	23.400	-1.503	-2.835	0.010	0.205	1
hsa-miR-584-3p	URS000006BE7D_9606	11.600	-1.230	-2.345	0.010	0.206	1
hsa-miR-3960	URS00003783AB_9606	3.000	2.104	4.300	0.013	0.247	1
hsa-miR-3667-3p	URS000075E2C3_9606	7.200	-1.722	-3.300	0.013	0.247	1
hsa-miR-148a-5p	URS00003E16E5_9606	88.000	-1.027	-2.038	0.014	0.248	1
hsa-miR-210-3p	URS000055128B_9606	156.400	-0.893	-1.856	0.014	0.248	1
hsa-miR-4707-3p	URS000018D2BE_9606	3.200	-2.125	-4.363	0.014	0.248	1
hsa-miR-95-3p	URS00002E2DE7_9606	22.308	1.294	2.451	0.014	0.248	1
hsa-miR-664b-5p	URS000042B108_9606	54.769	1.289	2.443	0.014	0.248	1
hsa-miR-1268b	URS000033B91D_9606	1.538	3.616	12.259	0.014	0.248	1
hsa-miR-1275	URS000009EA8F_9606	15.200	-1.967	-3.909	0.015	0.248	1
hsa-miR-9985	URS0000D4FAF7_9606	3.692	1.722	3.299	0.016	0.248	1
hsa-miR-18a-3p	URS00004131FE_9606	76.800	-1.016	-2.022	0.016	0.248	1
hsa-miR-676-3p	URS00004407DC_9606	1.615	4.312	19.862	0.016	0.248	1
hsa-miR-224-5p	URS0000D55DFB_9606	2664.154	1.093	2.133	0.016	0.248	1
hsa-miR-3614-3p	URS000042BE4B_9606	5.000	-1.553	-2.934	0.016	0.248	1
hsa-miR-182-5p	URS00001CC379_9606	870.385	0.834	1.783	0.016	0.248	1
hsa-miR-29b-3p	URS000024463E_9606	2805.000	-0.887	-1.849	0.017	0.256	1
hsa-miR-323a-3p	URS00003CCAB4_9606	172.769	0.965	1.951	0.017	0.256	1
hsa-miR-218-5p	URS000020D84A_9606	3.769	2.254	4.770	0.018	0.261	1
hsa-miR-363-3p	URS000038B599_9606	2360.200	-0.804	-1.746	0.020	0.283	1
hsa-miR-320e	URS00003B6B84_9606	3.615	2.068	4.193	0.020	0.283	1
hsa-miR-4646-5p	URS00001A6D0C_9606	2.385	2.142	4.413	0.020	0.283	1
hsa-miR-339-3p	URS000055B190_9606	710.200	-0.931	-1.906	0.021	0.283	1
hsa-miR-4449	URS00004DE2FC_9606	3.200	-1.613	-3.058	0.021	0.283	1
hsa-miR-6779-5p	URS000075A236_9606	2.692	1.973	3.927	0.021	0.283	1
hsa-miR-181a-2-3p	URS0000241987_9606	273.769	0.815	1.760	0.022	0.283	1
hsa-miR-4669	URS00001062A2_9606	1.538	4.216	18.586	0.022	0.283	1
hsa-miR-7705	URS000060B2B9_9606	7.800	-1.340	-2.531	0.023	0.283	1
hsa-miR-375-3p	URS00000ED600_9606	57.600	1.199	2.296	0.023	0.283	1
hsa-miR-320d	URS0000010C72_9606	134.600	1.016	2.023	0.023	0.283	1
hsa-miR-6796-5p	URS0000759E61_9606	2.692	2.454	5.478	0.023	0.283	1
hsa-miR-4786-5p	URS000023BDD3_9606	1.846	1.779	3.431	0.023	0.283	1
hsa-miR-484	URS0000597BED_9606	3301.800	-1.036	-2.051	0.024	0.295	1
hsa-miR-29c-5p	URS0000497496_9606	424.200	-0.835	-1.784	0.025	0.305	1
hsa-miR-548au-5p	URS000075CAF3_9606	73.600	-0.877	-1.837	0.027	0.326	1
hsa-miR-19a-3p	URS000006FDD4_9606	1111.400	-0.917	-1.889	0.028	0.328	1
hsa-miR-449a	URS00001F5B39_9606	23.200	-1.184	-2.273	0.028	0.328	1
hsa-miR-150-3p	URS00005EAAAD_9606	6.615	1.465	2.761	0.029	0.328	1

hsa-miR-3938	URS000075EB68_9606	1.400	-2.328	-5.021	0.029	#N/D	1
hsa-miR-2116-3p	URS00005237AB_9606	8.000	-1.437	-2.707	0.030	0.335	1
hsa-miR-6505-5p	URS000075D8DA_9606	4.154	1.375	2.593	0.030	0.339	1
hsa-miR-222-3p	URS00002C6949_9606	1930.600	-0.783	-1.720	0.031	0.339	1
hsa-miR-6751-3p	URS000075EDBE_9606	1.600	-1.995	-3.985	0.031	#N/D	1
hsa-miR-4767	URS000035D2F9_9606	2.800	-1.977	-3.935	0.032	0.349	1
hsa-miR-4454	URS00005D12AC_9606	305.400	-0.750	-1.681	0.033	0.351	1
hsa-miR-4446-3p	URS000000EF0B_9606	172.600	0.903	1.870	0.033	0.351	1
hsa-miR-3117-3p	URS0000507F3E_9606	2.000	-1.617	-3.068	0.034	0.351	1
hsa-miR-186-5p	URS000040DCFF_9606	5407.400	-0.723	-1.651	0.034	0.351	1
hsa-miR-151a-3p	URS000016C318_9606	33270.200	0.718	1.645	0.034	0.351	1
hsa-miR-491-5p	URS00001919B0_9606	300.600	-0.753	-1.685	0.035	0.353	1
hsa-miR-423-3p	URS00000BE495_9606	9811.600	-0.739	-1.669	0.036	0.353	1
hsa-miR-3657	URS000075C59B_9606	2.200	-1.744	-3.349	0.036	0.353	1
hsa-miR-28-5p	URS00003E47B1_9606	407.600	-0.745	-1.676	0.036	0.353	1
hsa-miR-664a-5p	URS0000259AE4_9606	461.200	0.871	1.829	0.036	0.353	1
hsa-miR-4785	URS0000266339_9606	16.400	-0.962	-1.949	0.036	0.353	1
hsa-miR-490-5p	URS00004556E5_9606	11.000	-1.422	-2.680	0.037	0.353	1
hsa-miR-671-5p	URS00002FB368_9606	311.000	-0.822	-1.768	0.038	0.358	1
hsa-miR-3200-3p	URS0000381B86_9606	3.538	1.531	2.891	0.038	0.358	1
hsa-miR-4662a-5p	URS00005140D0_9606	25.462	0.797	1.737	0.039	0.359	1
hsa-miR-19b-3p	URS000013D17D_9606	2734.200	-0.811	-1.755	0.039	0.359	1
hsa-miR-5582-3p	URS000075D126_9606	8.600	-1.359	-2.566	0.040	0.359	1
hsa-miR-206	URS000034B6F5_9606	11.154	1.904	3.743	0.041	0.359	1
hsa-miR-128-1-5p	URS0000537082_9606	10.200	1.156	2.228	0.041	0.359	1
hsa-miR-874-3p	URS00005609ED_9606	40.400	-0.808	-1.751	0.041	0.359	1
hsa-miR-4750-5p	URS0000210075_9606	11.538	1.195	2.290	0.041	0.359	1
hsa-miR-199a-5p	URS0000554A4F_9606	2061.200	-0.794	-1.734	0.042	0.359	1
hsa-miR-4433a-3p	URS00005A3BC9_9606	42.769	1.149	2.218	0.043	0.359	1
hsa-miR-485-5p	URS00001935FA_9606	242.600	0.862	1.818	0.043	0.359	1
hsa-miR-181b-3p	URS0000229622_9606	2.615	1.920	3.784	0.043	0.359	1
hsa-miR-4659a-3p	URS00003003B4_9606	8.400	-1.028	-2.039	0.043	0.359	1
hsa-miR-296-3p	URS00001F4670_9606	49.600	-1.047	-2.066	0.043	0.359	1
hsa-miR-10a-5p	URS000016D2D4_9606	801.077	0.664	1.585	0.043	0.359	1
hsa-miR-382-3p	URS000013E79D_9606	143.200	0.814	1.758	0.044	0.359	1
hsa-miR-3175	URS00002394F5_9606	1.615	2.273	4.833	0.044	0.359	1
hsa-miR-769-3p	URS00005F5472_9606	110.200	-0.868	-1.825	0.044	0.359	1
hsa-miR-2355-5p	URS0000609C67_9606	19.200	-1.054	-2.077	0.045	0.359	1
hsa-miR-16-1-3p	URS000061CB8F_9606	15.600	-1.079	-2.113	0.045	0.359	1
hsa-miR-4772-3p	URS00005901AD_9606	1.077	2.584	5.995	0.045	0.359	1
hsa-miR-24-2-5p	URS00001DEE11_9606	6.400	-1.563	-2.955	0.046	0.359	1
hsa-miR-503-5p	URS00000F6E49_9606	367.200	-0.791	-1.730	0.047	0.371	1
hsa-miR-597-5p	URS000075BC35_9606	2.000	-1.618	-3.070	0.048	0.372	1
hsa-miR-7109-5p	URS000075EDFB_9606	1.000	-2.693	-6.467	0.049	#N/D	1
hsa-miR-196b-3p	URS000052E1A6_9606	1.000	-2.693	-6.466	0.049	#N/D	1
hsa-miR-548g-3p	URS000075C7C4_9606	14.308	0.967	1.955	0.049	0.375	1

hsa-miR-4701-5p	URS000021FE20_9606	8.800	-0.917	-1.888	0.049	0.375	1
hsa-miR-577	URS00004CD810_9606	1.200	-2.133	-4.385	0.050	#N/D	1
hsa-miR-188-3p	URS000061AB33_9606	1.200	-2.132	-4.383	0.050	#N/D	1
hsa-miR-362-5p	URS0000085F64_9606	120.200	-0.760	-1.694	0.050	0.375	1

Differentially expressed miRNAs in AHI versus CHI with p-value< 0.05 are represented. Identifiers represent the code attributed to each miRNA according to RNA central database. Max group mean is the maximum of the average of reads per kilobase per million mapped reads (RPKM).

Supplementary Table S2. GO Enrichment Analysis (p-values< 0.05).

GO term	Description	DE Genes	DE Genes (Names)	P-values
0030947	regulation of vascular endothelial growth factor receptor signaling pathway	2	hsa-miR-296-5p, hsa-miR-10b-5p	0.004581
0030949	positive regulation of vascular endothelial growth factor receptor signaling pathway	2	hsa-miR-296-5p, hsa-miR-10b-5p	0.004581
0090287	regulation of cellular response to growth factor stimulus	3	hsa-miR-296-5p, hsa-miR-1-3p, hsa-miR-10b-5p	0.015032
0051272	positive regulation of cellular component movement	4	hsa-miR-296-5p, hsa-miR-1-3p, hsa-miR-1290, hsa-miR-10b-5p	0.01662
1903672	positive regulation of sprouting angiogenesis	3	hsa-miR-296-5p, hsa-miR-1-3p. hsa-miR-10b-5p	0.019807
0006928	movement of cell or subcellular component	1	hsa-miR-1-3p	0.030612
0010460	positive regulation of heart rate	1	hsa-miR-1-3p	0.030612
0010638	positive regulation of organelle organization	1	hsa-miR-1-3p	0.030612
0010640	regulation of platelet-derived growth factor receptor signaling pathway	1	hsa-miR-296-5p	0.030612
0010641	positive regulation of platelet-derived growth factor receptor signaling pathway	1	hsa-miR-296-5p	0.030612
0016477	cell migration	1	hsa-miR-1-3p	0.030612
0032411	positive regulation of transporter activity	1	hsa-miR-1-3p	0.030612
0032414	positive regulation of ion transmembrane transporter activity	1	hsa-miR-1-3p	0.030612
0035441	cell migration involved in vasculogenesis	1	hsa-miR-1-3p	0.030612
0040011	locomotion	1	hsa-miR-1-3p	0.030612
0045760	positive regulation of action potential	1	hsa-miR-1-3p	0.030612
0045823	positive regulation of heart contraction	1	hsa-miR-1-3p	0.030612
0045989	positive regulation of striated muscle contraction	1	hsa-miR-1-3p	0.030612
0048641	regulation of skeletal muscle tissue development	1	hsa-miR-1-3p	0.030612
0048643	positive regulation of skeletal muscle tissue development	1	hsa-miR-1-3p	0.030612
0048870	cell motility	1	hsa-miR-1-3p	0.030612
0051495	positive regulation of cytoskeleton organization	1	hsa-miR-1-3p	0.030612
0051928	positive regulation of calcium ion transport	1	hsa-miR-1-3p	0.030612
0060297	regulation of sarcomere organization	1	hsa-miR-1-3p	0.030612
0060298	positive regulation of sarcomere organization	1	hsa-miR-1-3p	0.030612
0060373	regulation of ventricular cardiac muscle cell membrane depolarization	1	hsa-miR-1-3p	0.030612

0060411	cardiac septum morphogenesis	1	hsa-miR-1-3p	0.030612
0060412	ventricular septum morphogenesis	1	hsa-miR-1-3p	0.030612
0060452	positive regulation of cardiac muscle contraction	1	hsa-miR-1-3p	0.030612
0060973	cell migration involved in heart development	1	hsa-miR-1-3p	0.030612
0060980	cell migration involved in coronary vasculogenesis	1	hsa-miR-1-3p	0.030612
0071875	adrenergic receptor signaling pathway	1	hsa-miR-1-3p	0.030612
0086023	adrenergic receptor signaling pathway involved in heart process	1	hsa-miR-1-3p	0.030612
0086094	positive regulation of ryanodine-sensitive calcium-release channel activity by adrenergic receptor signaling pathway involved in positive regulation of cardiac muscle contraction	1	hsa-miR-1-3p	0.030612
0086103	G-protein coupled receptor signaling pathway involved in heart process	1	hsa-miR-1-3p	0.030612
0090677	reversible differentiation	1	hsa-miR-1-3p	0.030612
1901018	positive regulation of potassium ion transmembrane transporter activity	1	hsa-miR-1-3p	0.030612
1902115	regulation of organelle assembly	1	hsa-miR-1-3p	0.030612
1902117	positive regulation of organelle assembly	1	hsa-miR-1-3p	0.030612
1903116	positive regulation of actin filament-based movement	1	hsa-miR-1-3p	0.030612
1903760	regulation of voltage-gated potassium channel activity involved in ventricular cardiac muscle cell action potential repolarization	1	hsa-miR-1-3p	0.030612
1903762	positive regulation of voltage-gated potassium channel activity involved in ventricular cardiac muscle cell action potential repolarization	1	hsa-miR-1-3p	0.030612
1903818	positive regulation of voltage-gated potassium channel activity	1	hsa-miR-1-3p	0.030612
1903947	positive regulation of ventricular cardiac muscle cell action potential	1	hsa-miR-1-3p	0.030612
1904427	positive regulation of calcium ion transmembrane transport	1	hsa-miR-1-3p	0.030612
1904879	positive regulation of calcium ion transmembrane transport via high voltage-gated calcium channel	1	hsa-miR-1-3p	0.030612
1905026	positive regulation of membrane repolarization during ventricular cardiac muscle cell action potential	1	hsa-miR-1-3p	0.030612
1905033	positive regulation of membrane repolarization during cardiac muscle cell action potential	1	hsa-miR-1-3p	0.030612
1905904	- No description available -	1	hsa-miR-1-3p	0.030612
1905913	- No description available -	1	hsa-miR-1-3p	0.030612
2001014	regulation of skeletal muscle cell differentiation	1	hsa-miR-1-3p	0.030612
2001016	positive regulation of skeletal muscle cell differentiation	1	hsa-miR-1-3p	0.030612
2001259	positive regulation of cation channel activity	1	hsa-miR-1-3p	0.030612

GO Term was the code associated with the biological processes. DE were the genes associated to differentially expressed miRNAs.
